# Visualizing the cellular route of entry of a cystine-knot peptide with Xfect transfection reagent by electron microscopy

**DOI:** 10.1038/s41598-019-43285-5

**Published:** 2019-05-06

**Authors:** Xinxin Gao, Ann De Mazière, David B. Iaea, Christopher P. Arthur, Judith Klumperman, Claudio Ciferri, Rami N. Hannoush

**Affiliations:** 1 Department of Early Discovery Biochemistry, Genentech, South San Francisco, California, USA; 2 Section of Cell Biology, Center for Molecular Medicine, University Medical Center Utrecht, Utrecht University, Utrecht, The Netherlands; 3 Department of Biochemical and Cellular Pharmacology, Genentech, South San Francisco, California, USA; 4 Department of Structural Biology, Genentech, South San Francisco, California, USA

**Keywords:** Peptides, Peptides, Cellular imaging, Drug delivery, Endocytosis

## Abstract

Cystine-knot peptides are attractive templates in drug discovery due to a number of features they possess including their 3D conformation, physicochemical stability and synthetic tractability. Yet, their cellular uptake mechanisms remain largely unexplored. Recently, we demonstrated that the cystine-knot peptide EETI-II is internalized into cells and that its cellular uptake could be modulated by using a protein transfection reagent Xfect. However, the mechanism of Xfect-mediated cellular internalization of EETI-II remained unclear. Here, by using high resolution electron microscopy, we observe the formation of EETI-II-positive macropinosomes and clathrin-coated pits at early time points after treatment of cells with EETI-II/Xfect complexes. Internalized EETI-II subsequently accumulates in intracellular Xfect-induced detergent-resistant membrane compartments which appear to lack characteristic endosomal or lysosomal markers. Notably, Xfect enables the uptake of cell impermeable nuclear dyes into similar intracellular compartments that do not seem to deliver the cargo to the cytosol or nucleus. Altogether, our findings reveal mechanistic insights into the cellular uptake route of Xfect, and underscore the need for the development of effective tools to enhance the cytosolic delivery of cystine-knot peptides. Finally, our data illustrate that electron microscopy is a powerful approach for studying endocytic mechanisms of cell-penetrating peptides and their effects on cellular membranes.

## Introduction

Cystine-knot peptides (CKP) have a highly constrained structure, comprising a knotted arrangement of three disulfide bonds^1^, which confers remarkable thermal, chemical and proteolytic stability^2^. These properties make CKPs attractive templates in drug discovery^3,4^. For instance, CKPs have been engineered to bind with good affinity to multiple extracellular proteins including growth factors, GPCRs and proteases, to name a few^5–12^. Antagonist CKPs that target vascular endothelial growth factor A (VEGF-A) were developed by grafting an arginine-rich peptide onto the kalata B1 framework. Anti-viral CKPs targeting foot-and-mouth viral 3C protease were generated using Momordica cochinchinensis trypsin inhibitor-II (MCoTI-II) as backbone^12,13^. Although the potency of the first generation inhibitors is moderate (low µM), the studies so far have demonstrated the feasibility of using CKPs as scaffolds for potential drug discovery applications. More recently, CKPs targeting proteins with antibody-like affinities have been engineered. For example, low nM affinity peptide antagonists that bound to cells expressing α_v_β_3_ integrins were identified by grafting the integrin recognition motif Arg-Gly-Asp onto Agouti-related protein (AgRP), a CKP with four disulfide bonds^9^. Other high affinity CKP antagonists to thrombopoietin, the primary regulator of platelet production, were also developed by grafting phage-derived peptides onto AgRP and Ecballium elaterium trypsin inhibitor-II (EETI-II) scaffolds^10^.

The potential of CKPs as pharmacological ligands has been expanded beyond extracellular protein targets. Engineered chimeric cystine-knot peptides incorporating a p53-targeting alpha helix, which was grafted onto loop 6 of the MCoTI-I framework, displayed low nM affinity to both Hdm2 and HdmX and exhibited stability in human serum and on-target cellular activity^14^. Furthermore, a potent CKP antagonist was designed against SET, a nuclear protein overexpressed in various human cancers, by grafting apolipoprotein E-derived COG peptides onto loop 6 of MCoTI-II framework. The engineered CKP demonstrated growth inhibitory effect in K562 cancer cell lines overexpressing SET, and inhibited NF-κB-dependent transcriptional responses in macrophages^5^. Although the cellular uptake route that these chimeric CKPs take to engage their intracellular targets remains unknown, the above studies provide early proof of concept about the potential utility of cystine-knot peptides for targeting cytosolic proteins.

Other work investigating the cellular uptake of wild-type CKPs demonstrated that they can enter cells through overlapping yet distinct mechanisms. For instance, MCoTI is internalized into mammalian cells primarily via macropinocytosis and, to a lesser extent, cholesterol-dependent and clathrin-mediated endocytosis, eventually accumulating in lysosomes^15,16^. Another CKP which belongs to the kalata B1 family enters cells through a combination of endocytic mechanisms and direct interaction with phosphatidylethanolamine on the plasma membrane^17–19^. More recently, our laboratory showed that acyclic cystine-knot peptide EETI-II, a member of the EETI family of squash trypsin inhibitors, is internalized into mammalian cells, ending up in early endosomes and subsequently late endosomes and lysosomes^20^. Although different cystine-knot peptides seem to exhibit distinct cellular uptake mechanisms, the ones studied thus far seem to ultimately accumulate in the endosomal and lysosomal compartments.

We have used the acyclic cystine-knot peptide EETI-II (peptide sequence, GCPRILMRCKQDSDCLAGCVCGPNGFCG) as a model system to study the feasibility of re-targeting cystine-knot peptides out of the endosomal/lysosomal pathways with protein transfection reagents. One such example of a protein transfection reagent is Xfect^20^, which is a cell-penetrating peptide of unknown composition that has been reported to bind to protein cargos through ionic pairing and deliver proteins into cells. For instance, it forms complexes with bovine serum albumin and transports it into mammalian cells or human hepatoma multicellular tumor spheroids^21,22^. It also enhances the cellular uptake of peptides such as EETI-II^20^. However, the cellular uptake mechanisms of EETI-II/Xfect, as well as the nature of the cellular compartments where cargos are targeted to by Xfect, are largely unknown. To address these questions, here we investigated the cellular trafficking pathways and localization of EETI-II/Xfect using high resolution electron microscopy (EM) and high-content fluorescence microscopy. We show that EETI-II/Xfect is internalized into mammalian cells via macropinocytosis and, in part, clathrin-mediated pathways. Internalized EETI-II/Xfect complex is targeted to cellular membrane compartments which seem to lack endosomal/lysosomal markers and are resistant to detergent extraction. These compartments are quite distinct from those to which EETI-II traffics by itself in the absence of Xfect. Importantly, using various cell penetrating protein/peptide and cell impermeable nuclear dyes, we demonstrate that Xfect delivers cell impermeable cargos into mammalian cells. Finally, studies performed in reconstituted liposomes revealed that Xfect influences membrane thickness and integrity.

## Results and Discussion

### Cellular uptake of rEETI-II-Alexa488/Xfect involves macropinocytic and clathrin-mediated pathways

**Figure 1:**
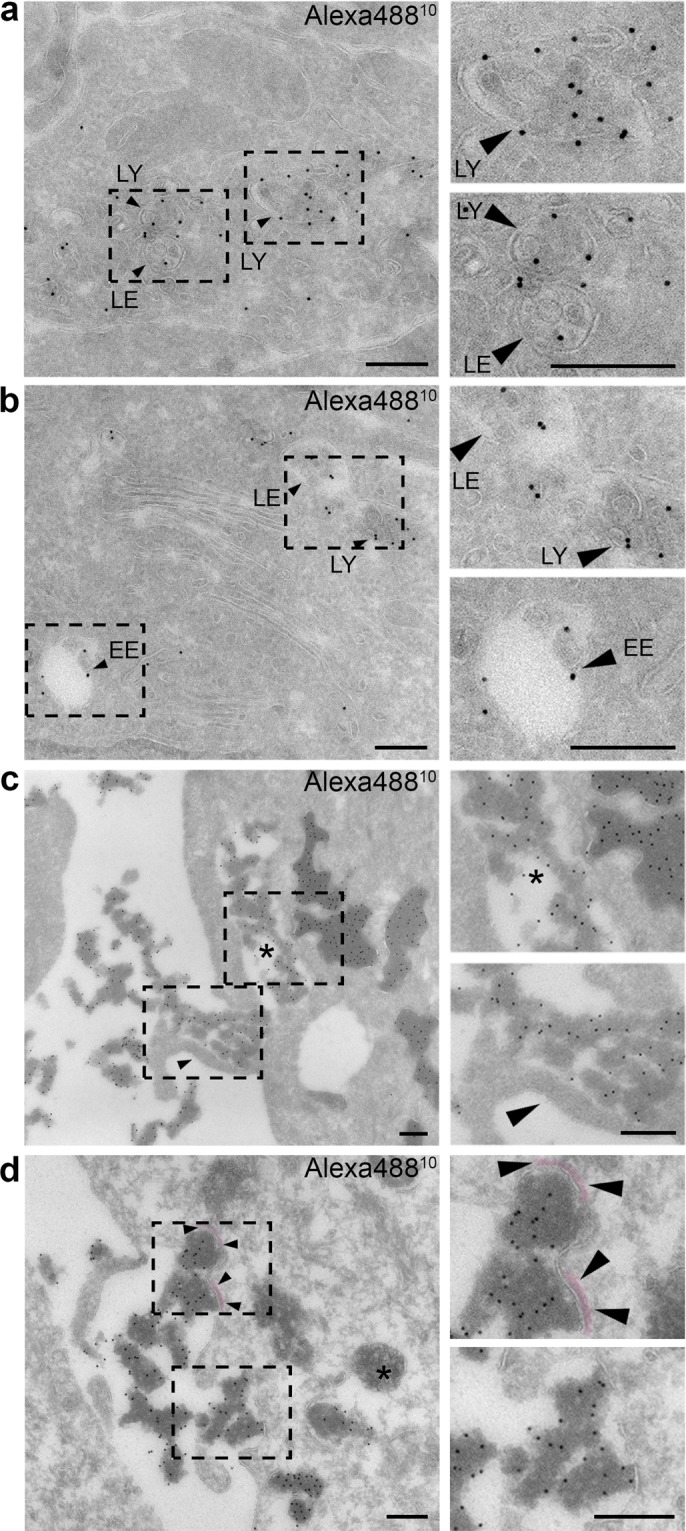
Electron microscopy of EETI-II/Xfect uptake into cells highlighting formation of macropinosomes and clathrin-coated plasma membrane invaginations. (**a**,**b**) Internalized EETI-II can be found in early endosomes, late endosomes and lysosomes. HeLa cells were treated with 50 uM rEETI-II-Alexa488 for 6 h and then fixed with PFA. EE: early endosome; LE: late endosome, LY: lysosome. (**c**) Electron microscopy image showing a microvillus (arrow) that engulfs rEETI-II-Alexa488/Xfect through macropinocytosis. Asterisk: forming/formed macropinosome. HeLa cells were treated with 5 μM rEETI-II-Alexa488/Xfect (rEETI-II: Xfect; 1:4) for 3 h and then fixed with PFA-GA as described in methods. (**d**) Electron microscopy image showing parts of the plasma membrane invaginations that engulf extracellular rEETI-II-Alexa488/Xfect being decorated with clathrin coats (shown in between each pair of arrowheads). The two clathrin coats are painted (false color) in pale, transparent pink. Only parts of the plasma membrane invaginations that engulf extracellular EETI-II-Alexa488-positive material are decorated with such a clathrin coat. Asterisk: MVB, negative for EETI-II-Alexa488. HeLa cells were treated with 5 μM rEETI-II-Alexa488/Xfect for 3 h and then fixed with PFA. Scale bar, 200 nm.

We utilized electron microscopy as an approach to examine the cellular uptake mechanism of rEETI-II-Alexa488/Xfect at high resolution. First, we established that, in the absence of Xfect, EETI-II-Alexa488 ends up in endosomes and lysosomes of HeLa cells as observed by electron microscopy (Fig. 1a,b), consistent with earlier data from fluorescence microscopy^20^. On the other hand, typical macropinocytic events were observed on the plasma membrane of cells that were treated with rEETI-II-Alexa488/Xfect. For example, profiles of long bent microvilli or membrane ruffles were seen next to deep irregular plasma membrane invaginations filled with flocculent rEETI-II-Alexa488/Xfect (Fig. 1c, Supplementary Fig. 1). In some samples, parts of the observed invaginated plasma membrane were decorated with a characteristic clathrin coat, suggesting a role for clathrin-mediated endocytosis in rEETI-II-Alexa488/Xfect internalization (Fig. 1d). Membrane-lined compartments directly below the plasma membrane, with a lumen comprising a mixture of electron-dense Alexa488-positive material and electron-lucent Alexa488-negative area were observed, likely indicative of recently formed macropinosomes and plasma membrane invaginations still in contact with the extracellular space (Fig. 1c,d, Supplementary Fig. 1). As a control, samples that were treated with Xfect alone also showed similar internalization phenotypes, with flocculent Xfect observed in macropinocytic membranes (Supplementary Fig. 2). These observations support the notion that rEETI-II-Alexa488/Xfect is internalized into cells predominantly via macropinocytosis and also, in part, via clathrin-mediated endocytosis, and that Xfect radically changes the distribution of EETI-II.

**Figure 2:**
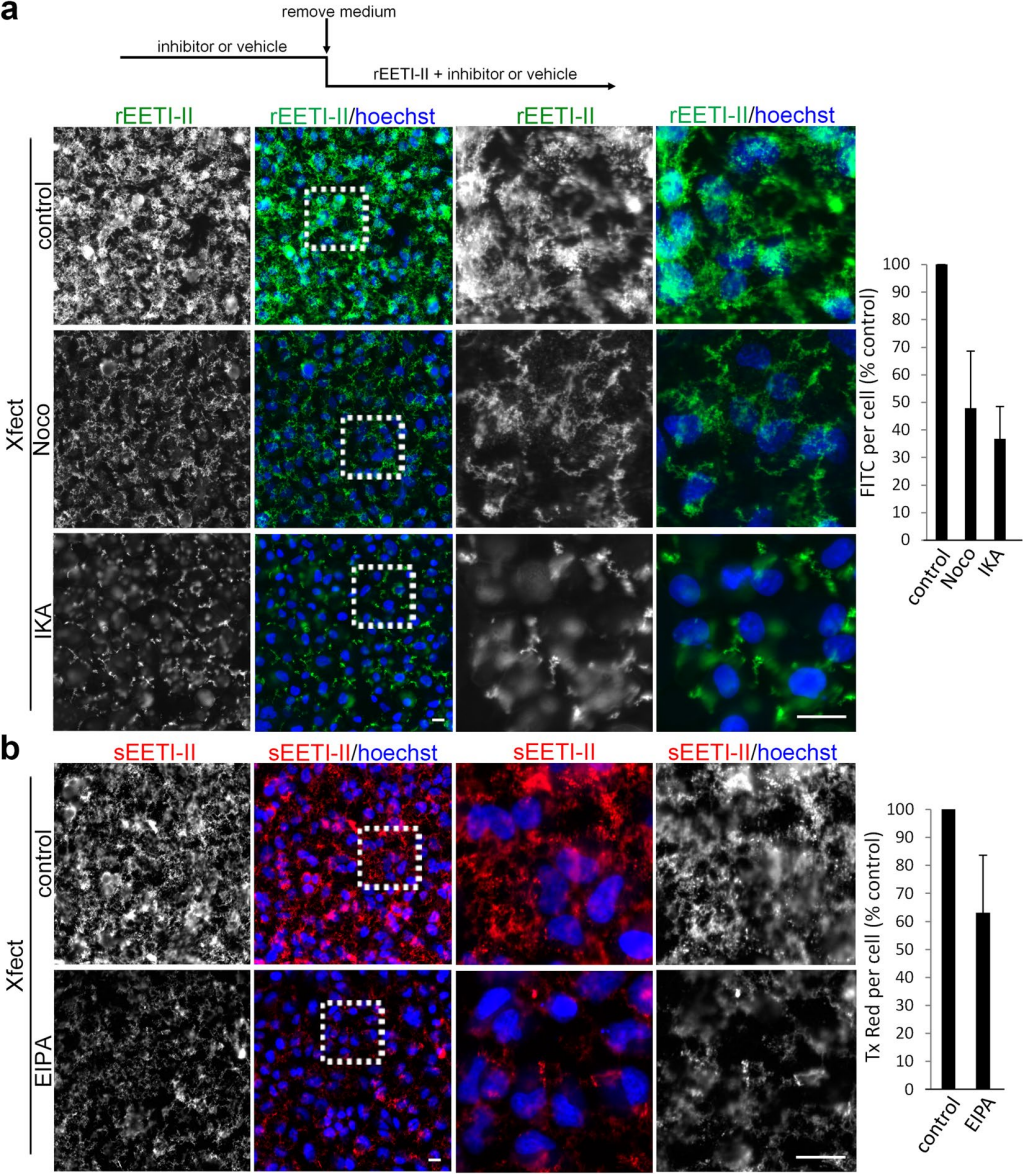
Cellular uptake of EETI-II/Xfect is diminished in the presence of nocodazole, ikarugamycin and EIPA. HeLa cells were treated with DMSO, nocodazole (10 μM), ikarugamycin (4 μM), or EIPA (50 μM) for 30 min, then with 5 μM rEETI-II-A488/Xfect (**a**) or sEETI-II-A594/Xfect (**b**) in the presence of DMSO or inhibitors for 60 min at 37 °C. Cells were washed with PBS then fixed with 4% PFA and processed as described in methods. Fluorescence images were captured on ImageXpress Micro XL imaging system (Molecular Devices) and analyzed by MetaXpress 4.0 (Molecular Devices). Fluorescence intensity values above a threshold defined using the DMSO-treated samples were measured and used to quantify integrated fluorescence intensity per cell. Values were normalized to the DMSO-treated samples and averaged from six independent experiments. Mean ± SD. n = 5500 cells. Representative images from at least six independent experiments are shown. Scale bar, 20 μm.

To complement the above results from electron microscopy, we evaluated the cellular uptake of rEETI-II-Alexa488/Xfect in the presence of endocytosis inhibitors by fluorescence microscopy. High-content imaging provides statistical power for both quantitative and qualitative analysis and is therefore a robust tool to validate EM findings. In this context, HeLa cells were pre-treated with inhibitors of clathrin-mediated endocytosis (ikarugamycin, IKA^23^, 4 μM) or macropinocytosis (ethylisopropyl amiloride, EIPA^24^, 50 μM) for 30 min. rEETI-II-Alexa488/Xfect was then added to the cells in the presence of the same inhibitors for 1 h. Due to the high fluorescence background of EIPA in the FITC channel, we used a red-shifted fluorophore to label synthetic EETI-II (with Alexa594) for experiments with EIPA treatment. Cellular uptake of rEETI-II-Alexa488/Xfect was inhibited by ~60% with IKA treatment, presumably due to inhibition of clathrin-mediated endocytosis (Fig. 2a). Similarly, the inhibitor for macropinocytosis, EIPA, moderately inhibited the uptake of sEETI-II-Alexa594/Xfect (~40%) (Fig. 2b). It is noteworthy that both internalized EETI-II-Alexa488 and EETI-II-Alexa594 exhibited similar phenotype, indicating that the nature of the fluorophore had minimal effect on the subcellular distribution of EETI-II. Moreover, treatment with nocodazole (10 μM), an inhibitor of microtubule polymerization^25^, diminished the internalization of rEETI-II-Alexa488/Xfect (Fig. 2a), indicating microtubules play important roles in transporting rEETI-II-Alexa488/Xfect from cell membrane to cellular vesicles. Previous reports have shown that treatment of cell monolayers or multicellular tumor spheroids with amiloride inhibited uptake of BSA/Xfect complexes^21,22^. Therefore, it’s possible that EETI-II/Xfect and BSA/Xfect complexes adopt overlapping cellular uptake routes. In control experiments, IKA, EIPA and nocodazole treatments also inhibited cellular uptake of EETI-II alone (Supplementary Figs 3–4), consistent with our previous finding that EETI-II is internalized via clathrin-mediated and macropinocytosis pathways^20^. These results confirm the above EM observations, and reveal that the trafficking of rEETI-II-Alexa488/Xfect is dependent on microtubules.

### Internalized rEETI-II-Alexa488/Xfect is targeted to membrane compartments which appear to lack endosomal/lysosomal markers

**Figure 3:**
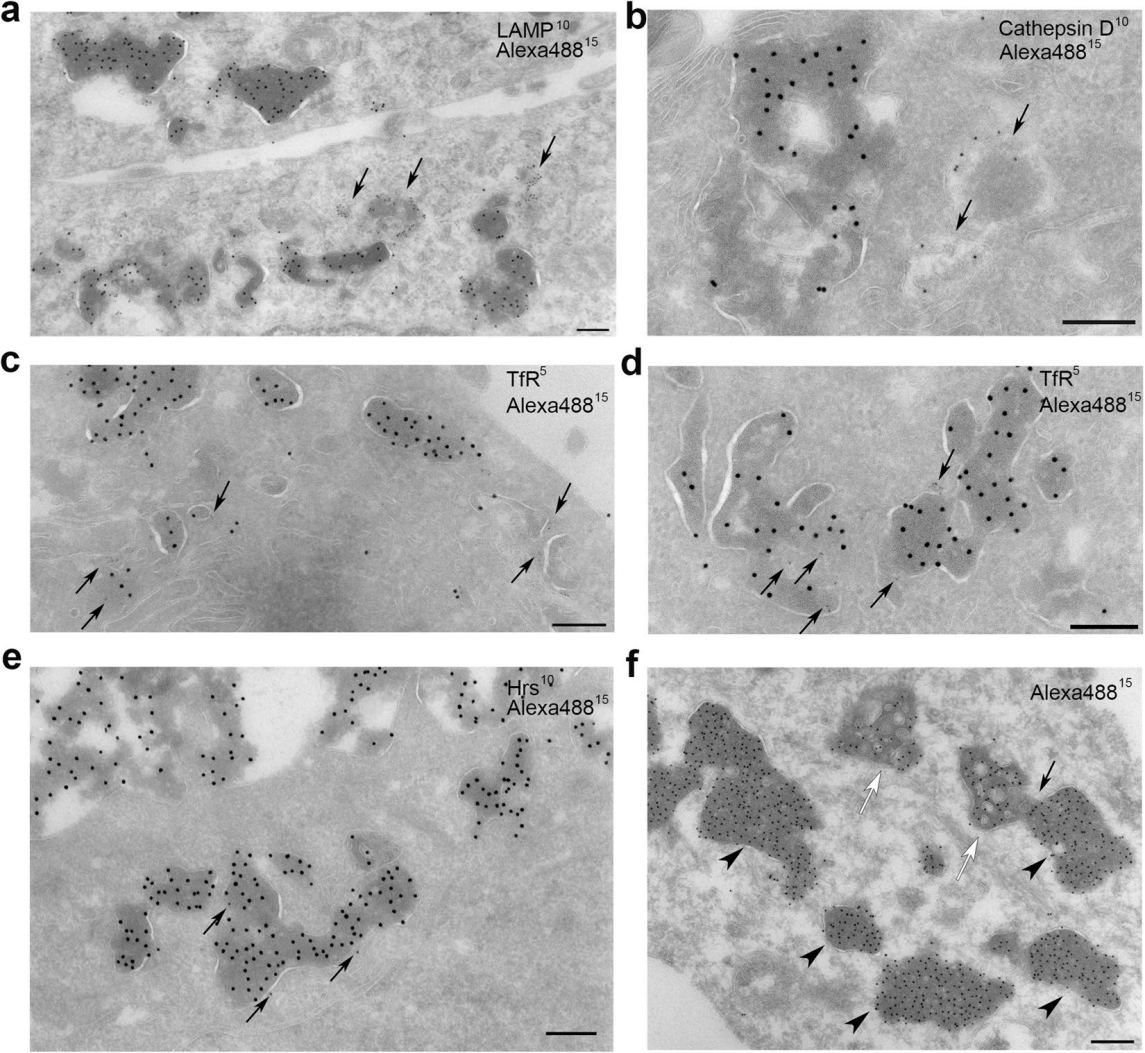
Electron microscopy images highlighting the presence of internalized EETI-II/Xfect within intracellular compartments devoid of endosomal and lysosomal markers. (**a**) Double labeling of LAMP-1 (10 nm gold particles) and Alexa488 (15 nm gold particles) shows that the majority of rEETI-II-Alexa488/Xfect positive compartments are LAMP-1 negative. Arrows: lysosomes. HeLa cells were treated with 5 μM rEETI-II-Alexa488/Xfect for 3 h then fixed with PFA. (**b**) Double labeling of cathepsin D (10 nm gold particles) and Alexa488 (15 nm gold particles) shows that the majority of rEETI-II-Alexa488/Xfect positive compartments are cathepsin D negative. Arrows: lysosomes. HeLa cells were treated with 5 μM rEETI-II-Alexa488/Xfect for 3 h then fixed with PFA-GA. (**c**,**d**) Double labeling of transferrin receptor (TfR, 5 nm gold particles) and Alexa488 (15 nm gold particles) shows that the majority of rEETI-II-Alexa488/Xfect positive compartments are transferrin receptor-negative. Arrows: Transferrin receptor label. HeLa cells were treated with 5 μM rEETI-II-Alexa488/Xfect for 30 min then fixed with PFA-GA as described in methods. (**e**) Double labeling of hepatocyte growth factor-regulated tyrosine kinase substrate (Hrs, 10 nm gold particles) and Alexa488 (15 nm gold particles) shows that the majority of rEETI-II-Alexa488/Xfect positive compartments are Hrs-negative. Arrows: Hrs label. HeLa cells were treated with 5 μM rEETI-II-Alexa488/Xfect for 30 min then fixed with PFA-GA. (**f**) Internalized rEETI-II-Alexa488/Xfect can be found in cellular compartments including multivesicular body-like compartments (open arrowheads). Solid arrowhead: Commonly found EETI-II/Xfect positive cellular compartments of unknown nature; arrow: point of fusion between MVB-like compartment and common EETI-II/Xfect positive compartment. HeLa cells were treated with 5 μM rEETI-II-Alexa488/Xfect for 3 h then fixed with PFA. Scale bar, 200 nm.

To further examine the nature of the compartments where rEETI-II-Alexa488/Xfect resides, we studied the colocalization pattern between internalized rEETI-II-Alexa488/Xfect and various endogenous protein markers in native cells using high resolution EM. We observed that the majority of cellular rEETI-II-Alexa488/Xfect is located in membrane-lined compartments, with little or no detectable rEETI-II-Alexa488/Xfect in the cytosol. Several markers for endosomes and lysosomes were probed to investigate the identity of these vesicles. Images from samples double labeled with Alexa488 and LAMP-1, a lysosomal membrane marker, revealed that all Alexa488-positive compartments appeared to lack LAMP-1 labeling (Fig. 3a). Similarly, ~90% of rEETI-II-Alexa488-positive compartments showed no labeling for cathepsin D, a soluble lysosome marker, in samples double labeled with Alexa488 and cathepsin D (Fig. 3b).

To examine whether the observed Alexa488-positive compartments had early endosomal characteristics, rEETI-II-Alexa488/Xfect treated samples were double labeled for Alexa488 and two endosome markers, Hrs (hepatocyte growth factor-regulated tyrosine kinase substrate) or transferrin receptor (TfR). Only a minority of the dense Alexa488-positive compartments displayed Hrs or transferrin receptor labeling at their limiting membrane (Fig. 3c–e). Roughly 80~90% of Alexa488-positive compartments were negative for transferrin receptor labeling. These Xfect-characteristic cellular compartments are newly formed upon Xfect incubation as supported by the observations that Xfect alone can induce the formation of such unique compartments (Supplementary Fig. 2), and no such compartments can be found in cells incubated with rEETI- II-Alexa488 alone (Fig. 1a,b). Interestingly, in some cells, we occasionally detected a small population of rEETI-II-Alexa488/Xfect residing in multivesicular body-like compartments (MVB-like compartments)^26^ (Fig. 3f). These MVB-like compartments differed from typical late endosomes or MVBs (e.g. in Fig. 1d, right lower corner) by their heterogeneously sized intraluminal vesicles and their irregular overall contour. The observations on the EETI-II-Alexa488/Xfect compartments are in sharp contrast to the subcellular distribution of internalized rEETI-II-Alexa488, which shows distinct endosome- and lysosome-specific cellular localization in the absence of Xfect co-incubation (Fig. 1a,b). Collectively, these findings establish that internalized rEETI-II-Alexa488/Xfect is present in distinct intracellular membrane compartments, lacking characteristic endosomal/lysosomal markers, that appear not to fuse with the endocytic pathway, and some rEETI-II-Alexa488/Xfect can enter MVB-like compartments. The origin of these membranes is not clear, but they seem to form only upon Xfect treatment.

### Internalized rEETI-II-Alexa488/Xfect resides in cellular membranes which are resistant to detergent extraction

We further characterized the Xfect-characteristic compartments using detergent-based cell permeabilization assays. Detergents such as Triton X-100, saponin and digitonin are routinely used to study intracellular membrane trafficking. Triton X-100 is a non-selective membrane permeabilization reagent with uncharged, hydrophilic head groups consisting of polyethylene oxide moieties. Saponin, a plant glycoside, and digitonin, a steroidal saponin, extract membrane cholesterol thus forming holes in the membrane. The plasma membrane contains a relatively higher concentration of cholesterol than the endosomal membrane. Hence the plasma membrane can be selectively permeabilized by using digitonin and low concentration of saponin, without compromising the integrity of certain intracellular membrane compartments such as endosomes^27,28^.

**Figure 4:**
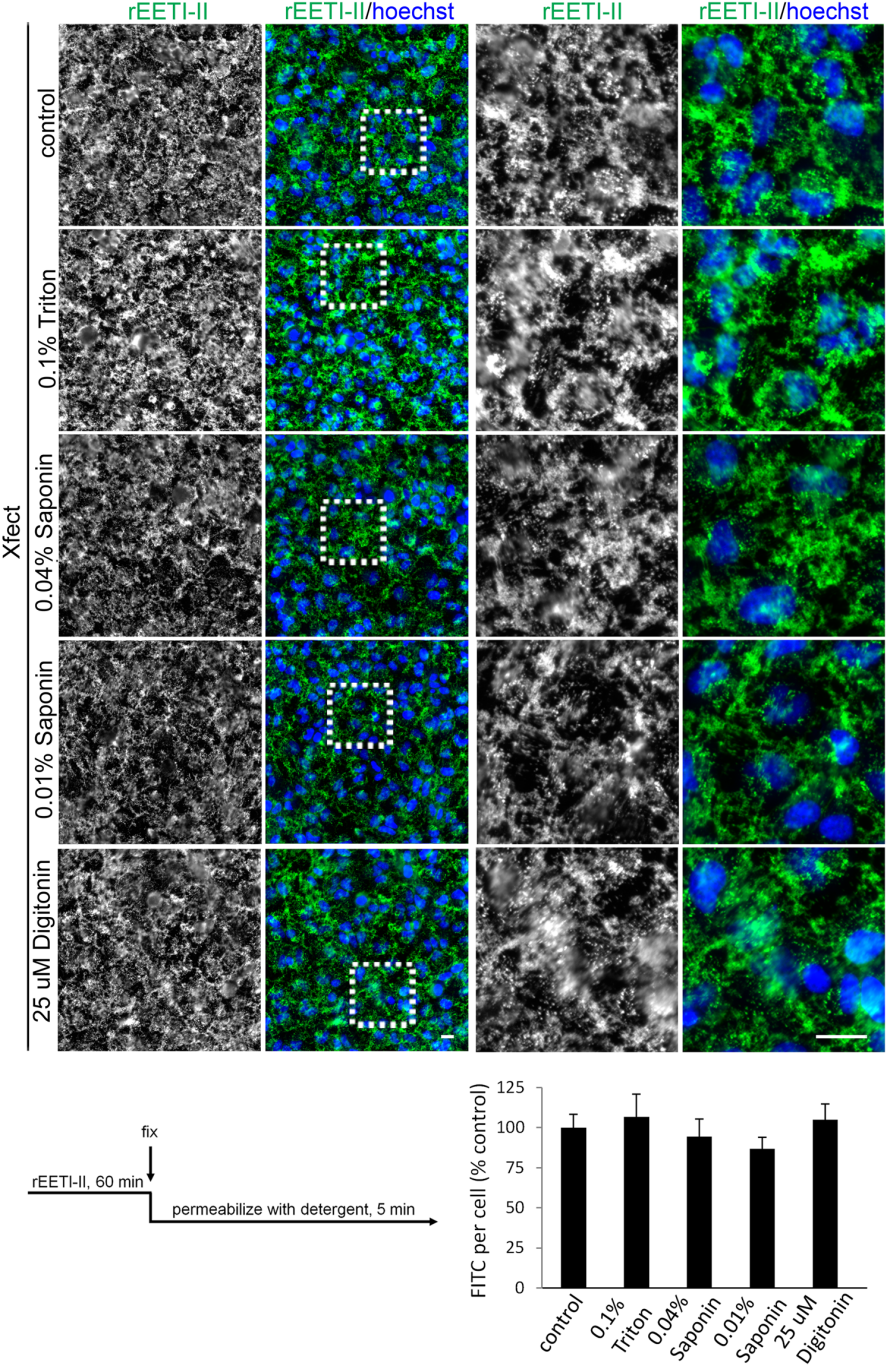
Internalized rEETI-II/Xfect is found in compartments that are resistant to detergent permeabilization. HeLa cells were treated with 5 μM rEETI-II-Alexa488/Xfect for 60 min then fixed with 4% PFA as described in methods. Cells were then incubated with PBS, 0.1% Triton X-100, 0.04% Saponin, 0.01% Saponin, or 25 μM Digitonin in PBS for 5 min at room temperature, washed and imaged. Fluorescence images were captured and analyzed as described in Fig. 2. Mean ± SD. n = 1000 cells. Representative images from at least three independent experiments are shown. Scale bar, 20 μm.

HeLa cells were incubated with either rEETI-II-Alexa488 or rEETI-II-Alexa488/Xfect and fixed with paraformaldehyde (PFA), then incubated with PBS, 0.1% Triton X-100, 0.04% or 0.01% saponin, or 25 µM digitonin for 5 min at room temperature. We observed that permeabilization with Triton X-100 or high concentration of saponin caused complete loss of internalized rEETI-II-Alexa488 signal, while low concentration of saponin treatment preserved ~50% of cellular rEETI-II-Alexa488 signal (Supplementary Fig. 5), due to full or partial permeabilization of endosomal/lysosomal membranes, respectively. Under milder permeabilization conditions using digitonin, in which the endosomal/lysosomal membrane structures were not compromised, the intensity and distribution pattern of internalized rEETI-II-Alexa488 was not affected (Supplementary Fig. 5). In sharp contrast, cellular staining of rEETI-II-Alexa488/Xfect was not altered by any of the above detergent permeabilization conditions, regardless of whether the integrity of endosomal/lysosomal membranes was compromised (Fig. 4). As a control experiment, fluorescently-labeled transferrin ligand, an established marker of early and recycling endosomes, was observed to be resistant to detergent extraction (Supplementary Fig. 6), due to the nature of internalized transferrin being bound to its receptor and hence not freely soluble. Together, these findings indicate that EETI-II is present freely soluble in the lumen of endosomes and lysosomes and, upon treatment with Xfect, is found in detergent-resistant membrane compartments. The findings that EETI-II cannot be extracted from these compartments by detergents suggest that it is tightly associated with X-fect-induced membranes, consistent with the EM observations.

### Xfect delivers cell-impermeable dyes to cells

**Figure 5:**
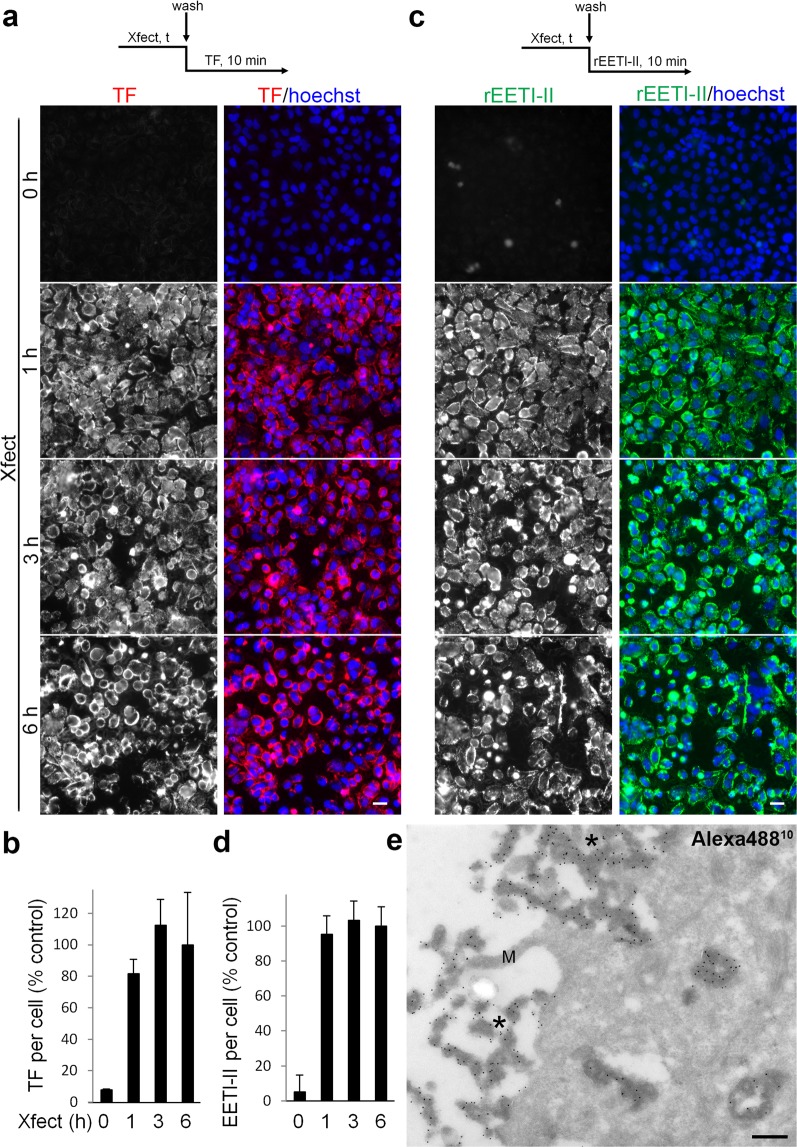
Internalized rEETI-II or transferrin is targeted to Xfect-induced membrane compartments in HeLa cells. HeLa cells were incubated with Xfect for indicated times then washed with PBS for three times and incubated with 0.2 mg/ml transferrin-Alexa555 (**a**,**b**) or 5 μM rEETI-II-Alexa488 (**c**,**d**) for 10 min, and washed with PBS again. Cells were fixed with 4% PFA and processed as described in methods. Fluorescence images were captured and analyzed as described in Fig. 2. Values were normalized to the 6 h Xfect treated samples. Mean ± SD. n = 1000 cells. Representative images from three independent experiments are shown. Scale bar, 20 µm. (**e**) EETI-II-ALexa488/Xfect is not washed away from the plasma membrane. Cells were incubated with 50 µM EETI-II-Alexa488/Xfect for 3 h, and then washed extensively with PBS before PFA fixation. The extracellular flocculent mixture of EETI-II-Alexa488 and Xfect (asterisks) sticks firmly to the cells after washing. M: microvillus. Scale bar, 200 nm.

**Figure 6:**
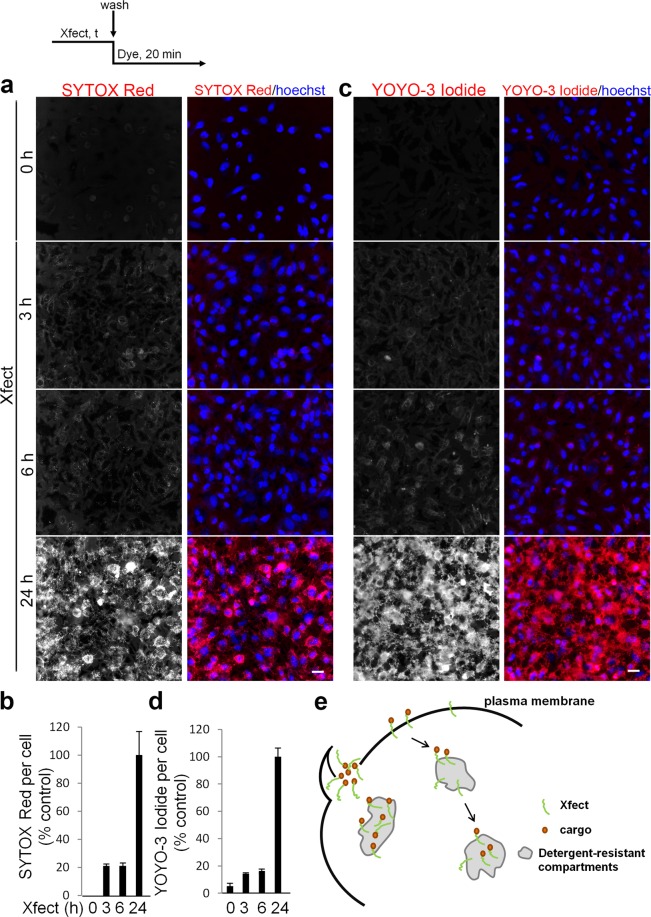
Xfect facilitates the delivery of cell impermeable dyes into HeLa cells. (**a**–**d**) HeLa cells were incubated with Xfect for indicated times then washed with PBS for three times and incubated with 30 nM SYTOX Red (**a**,**b**, Cy5 channel) or 200 nM YOYO-3 Iodide (**c**,**d**, Texas Red channel) for 20 min, and washed with PBS again. Cells were fixed with 4% PFA and processed as described in methods. Fluorescence images were captured and analyzed as described in Fig. 2. Values were normalized to the 24 h Xfect treated samples. Mean ± SD. n = 1000 cells. Representative images from three independent experiments are shown. Scale bar, 20 µm. (**e**) Proposed model of Xfect cellular entry and delivery of cargo. Xfect enables the delivery of protein, peptide, or small molecule cell impermeable nuclear dyes into Xfect-induced detergent-resistant membrane compartments inside mammalian cells through macropinocytosis.

To further understand the scope of Xfect-mediated uptake, we evaluated the effect of Xfect on the uptake of different cargo including, a protein (transferrin), a peptide (EETI-II) or small molecule cell impermeable dyes, in cells that were pre-treated with Xfect. HeLa cells were treated with Xfect for varying times then washed extensively to get rid of free Xfect, followed by incubation with rEETI-II-Alexa488 (5 µM, 10 min), transferrin-Alexa555 (0.2 mg/ml, 10 min), SYTOX Red (30 nM, MW = ~450 Da, 20 min) or YOYO-3 Iodide (200 nM, MW = 1322.7 Da, 20 min). For both rEETI-II-Alexa488 and transferrin-Alexa555, the internalization capacity has been greatly enhanced after preincubation of cells with Xfect (Fig. 5). The cell impermeable dyes SYTOX Red and YOYO-3 Iodide were observed to enter into cells that were pre-treated with Xfect for 3 h (Fig. 6a–d, Supplementary Fig. 7). As a negative control, the same small molecule dyes showed little to no uptake into cells in the absence of Xfect preincubation, as expected due to their cell impermeable nature (Fig. 6a,c). Interestingly, the majority of cellular SYTOX Red and YOYO-3 Iodide were found in compartments exclusive of the nucleus (Fig. 6a–d, Supplementary Fig. 7). After a 24 h preincubation period of cells with Xfect, the internalized dyes displayed a cellular distribution pattern that was reminiscent of what we observed earlier with rEETI-II-Alexa488/Xfect compartments. In fact, co-localization was observed between rEETI-II-Alexa488/Xfect and SYTOX Red/YOYO-3 Iodide in cells simultaneously treated with these reagents (Supplementary Fig. 8), indicating that these cargos were eventually delivered to Xfect-specific cellular compartments. Moreover, electron microscopy studies revealed that rEETI-II-Alexa488/Xfect is not simply washed away from the plasma membrane even after extensive washing before cell fixation (Fig. 5e). It is noteworthy that treatment of cells with Xfect for 24 h did not affect the total cell number (as detected by nuclear staining) and no major changes in nuclear morphology were noted, indicating that there is little or no cellular toxicity in the presence of Xfect. Together, the above findings suggest that treatment with Xfect leads to the formation of irregular membrane compartments to which different cargos can be delivered (Fig. 6e). It is unlikely that the Xfect-induced macropinosomes comprise a productive compartment for cytosolic delivery of cargo, since there is no cytoplasmic or nuclear cargo observed in our study. We hypothesize that the Xfect-induced membranes are simply long-lived macropinosomes that fail to traffic due to being clogged with unnatural flocculates of transfection reagent.

### Xfect, but not EETI-II, alters the integrity of liposome membranes

**Figure 7:**
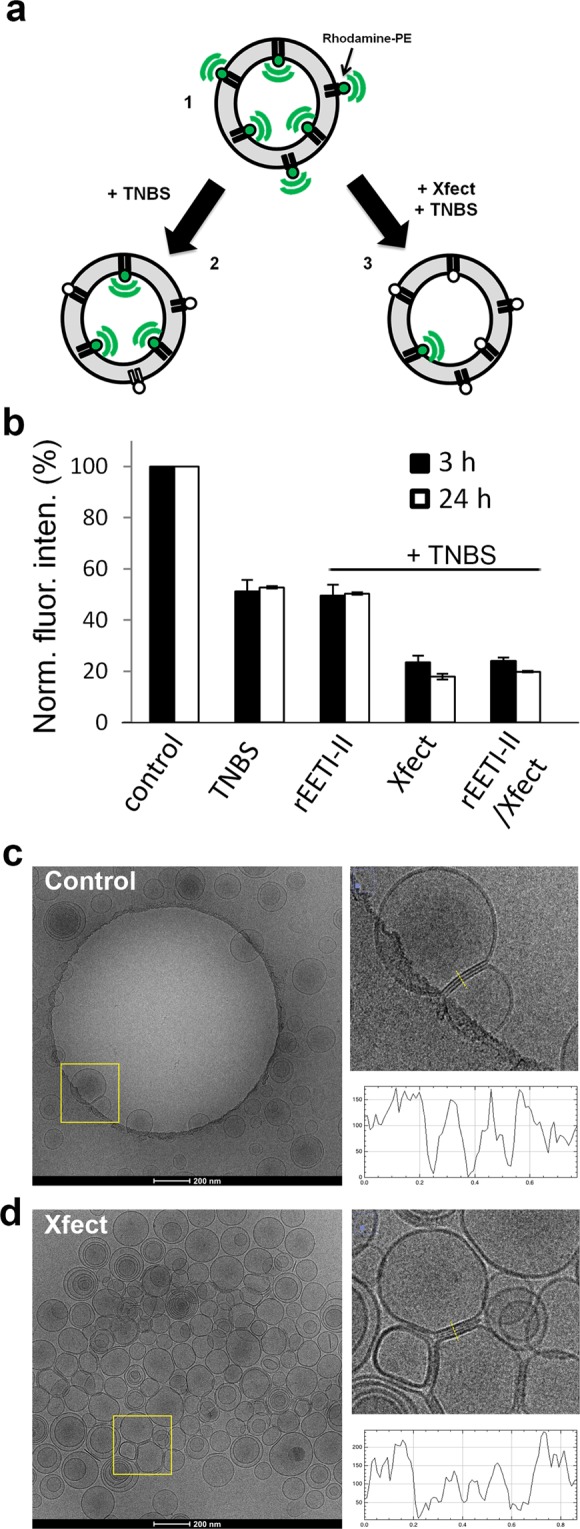
Xfect, but not EETI-II, affects the integrity of reconstituted liposome membranes. (**a**) Schematic of rhodamine-PE quenching. 1, Rhodamine-PE containing liposomes are prepared to generate symmetric membrane leaflets. 2, Addition of membrane impermeable, collisional quencher TNBS to the medium quenches rhodamine-PE fluorescence on the exofacial leaflet of the liposome. 3, Incubation with Xfect peptide facilitates TNBS crossing the bilayer to quench both the luminal and exofacial rhodamine-PE molecules. (**b**) Liposomes (100 μM) were pre-incubated in the absence (control) or presence of either rEETI-II (5 µM), Xfect (8% (v/v)) or both for either 3 or 24 h at room temperature with gentle agitation while protected from light. At the end of the incubation period, 25 mM TNBS was added to the mixture and fluorescence was measured at 560 nm/580 nm (excitation/emission) by using an EnSight reader. The fluorescence signal from wells treated with rEETI-II, Xfect or both were normalized to untreated control liposomes. Mean ± SEM. Data represent the average of two independent experiments. (**c**,**d**) Representative cryo-EM images of (**c**) untreated control liposomes or (**d**) Xfect-treated liposomes for 24 h. Enlarged boxed areas are shown as depicted in panels (c,d). The graphs illustrate line plot profiles of areas in between membranes, highlighting changes in the ultrastructure of the membrane in the presence of Xfect. Scale bar, 200 nm.

To further understand the effect of Xfect on the plasma membrane, we investigated the interaction between Xfect and reconstituted membrane bilayers in an *in vitro* liposome assay^29^. Symmetric liposomes containing equal amounts of rhodamine-phosphatidylethanolamine (rhodamine-PE) in each leaflet were prepared. We then examined if the addition of either rEETI-II or Xfect facilitates delivery of the membrane impermeable, collisional quencher, 2,4,6-trinitrobenzensulfonic acid (TNBS), across the lipid bilayer, resulting in quenching of the protected luminal rhodamine-PE (Fig. 7a). As expected, addition of TNBS to rhodamine-PE liposomes quenched approximately 50% of the fluorescence signal compared to non-treated liposomes (Fig. 7b). This finding is consistent with quenching of only the exofacial leaflet portion of rhodamine-PE, and demonstrates that the internal portion of the rhodamine-PE lipid is protected from TNBS quenching (Fig. 7a).

Liposomes (100 μM) were incubated with either 5 μM rEETI-II, 8% (v/v) Xfect or both for 3 or 24 h, and then quenched by addition of TNBS. Incubation with rEETI-II only did not alter the fluorescence signal, indicating that the luminal portion of rhodamine-PE remained protected. In contrast, pre-incubation with Xfect further decreased the fluorescence signal of rhodamine-PE lipids, presumably due to additional quenching of luminal rhodamine-PE by TNBS that is delivered inside liposomes by Xfect (Fig. 7b). It is noteworthy that Xfect treatment led to only 50–60% reduction in intrafacial leaflet rhodamine-PE fluorescence signal, and there was no increase in fluorescence quenching between 3 and 24 h incubations. A likely reason for the observed incomplete quenching is that the amount of TNBS effectively transduced through the artificial lipid membrane by Xfect is not sufficient to fully quench luminal rhodamine-PE. Moreover, it is conceivable that, in binding to membranes, Xfect may cause shielding of the lipid head groups, thereby protecting a portion of the luminal fluorophores from TNBS quenching and accounting for the observed remnant fluorescence signal.

Pre-incubation of both rEETI-II and Xfect did not introduce further quenching compared to Xfect-treated samples (Fig. 7b), suggesting that Xfect is responsible for delivering TNBS into the lumen of reconstituted liposomes. The results here are consistent with the above cellular data revealing Xfect-mediated delivery of cell impermeable cargos into mammalian cells, possibly by influencing the integrity of the membrane. Indeed, cryo-EM of liposomes demonstrated that Xfect treatment changed the ultrastructure of lipid membranes and promoted membrane fusions (Fig. 7c,d). The liposome membranes appeared as if they are stitched together and thicker than untreated membranes (Fig. 7d), suggesting that Xfect could exhibit a scaffolding role in controlling membrane thickness and permeability.

## Conclusion

Here, we utilized electron microscopy to delineate the different stages of cellular uptake of rEETI-II-Alexa488 mediated by the transfection reagent Xfect. Our EM data illustrated that Xfect can deliver rEETI-II-Alexa488 into mammalian cells primarily through macropinocytosis and, to a lesser extent, clathrin-mediated pathways, and this could be perturbed by endocytic inhibitors. Further observations based on EM, coupled with detergent permeabilization assays, established that the peptides delivered by Xfect are bounded by membrane-lined intracellular compartments that are resistant to detergent extraction and seem to lack characteristic endosome and lysosome markers. This phenotype is in sharp contrast to the intracellular distribution of rEETI-II-Alexa488 alone, which depicts accumulation in the lumen of endosomes and lysosomes. Notably, our studies showed that Xfect also delivers cell impermeable nuclear dyes into similar detergent-resistant compartments in mammalian cells.

The ability of Xfect to modulate membrane thickness and promote membrane fusion, as observed by cryo-EM, could be a potential mechanism by which Xfect influences endomembrane integrity leading to formation of X-fect macropinosomes. Interestingly, Xfect has been used to deliver large proteins such as BSA into cells^21,22^. The Xfect internalization routes of different cargo, including rEETI-II-Alexa488, BSA and small molecule cell impermeable dyes, seem to have some degree of overlap, and span clathrin- and caveolae-mediated endocytosis as well as macropinocytosis. However, it remains unclear whether variations in uptake mechanisms could also stem from differences in the physicochemical properties of the cargo, such as molecular weight, surface charge, or shape. For instance, previous attempts to deliver the small molecule X-Gal (5-bromo-4-chloro-3-indolyl-β-D-galactoside) using Xfect into MC3T3-E1.4 cells failed^30^. However, a FITC-labeled antibody was successfully delivered into the same cells by Xfect^30^, suggesting that compatibility between the cargo and Xfect might influence delivery.

Although the intracellular delivery of EETI-II is enhanced in the presence of Xfect, there is little or no cytosolic signal observed, raising a question about the utility of Xfect to deliver CKPs to the cytosol and arguing for the development of more effective peptide delivery tools. For instance, efforts to engineer cystine-knot peptides that get delivered to the cytosol are warranted. Finally, our findings underscore the value of using electron microscopy in the study of endocytic mechanisms of cell-penetrating peptides and in depth characterization of their effects on cellular membranes. This approach should prove useful in advancing the field of cellular peptide delivery forward.

## Methods

### Cell culture

HeLa cells (ATCC no. CCL-2) were grown in high glucose Dulbecco’s Modified Eagle’s Medium (DMEM) supplemented with 10% FBS and 2 mM Glutamax™. All cells were incubated in a 5% CO_2_ humidified incubator at 37 °C for 24 h before experiments. Cells were seeded onto Corning CellBIND 96 well plates (10,000 cells per well) and were grown for 24 h at 37 °C/5% CO_2_ before experiments.

### Fluorescence labeling of EETI-II

EETI-II was purified and labeled as described before^20^. Purified folded EETI-II (peptide sequence, GCPRILMRCKQDSDCLAGCVCGPNGFCG) was labeled with NHS-AlexaFluor488 or NHS-AlexaFluor594 (Invitrogen). Excess free dye and salt were removed by Sep-Pak C18 cartridge (Waters, cat # WAT043345). Labeled EETI-II was lyophilized, reconstituted in 50% DMSO (in H_2_O) and purified with a C18 reversed phase HPLC column. The final product (dye labeled EETI-II) was confirmed by mass spectrometry using a LC-MS system (Agilent Technologies) and comprises a mixture of single- and dual-labeled rEETI-II (at lysine K10 residue and the N terminus)^20^.

### Preparation of rEETI-II-Alexa488/Xfect complex

rEETI-II-A488 was incubated with the cell-penetrating peptide (Xfect™ Protein Transfection Reagent; Clontech, cat# 631324/rEETI-II-A488: Xfect; 1 µg: 4 µl) following the manufacturer’s protocol. Briefly, 16 µl of 1X Xfect protein transfection reagent stock solution (or deionized water only for control experiments with rEETI-II-A488 alone) were used to prepare complexes containing 4 µg of rEETI-II-A488 (final volume 100 µl; final concentration 10 µM). The complexes were incubated at room temperature for 30 min. To treat cells with complexes containing 5 µM rEETI-II-A488, 50 µl of the mixture was added to cells with 50 µl Opti-MEM^20^. Cells were incubated for the indicated times then processed for imaging.

### Cellular uptake assays of EETI-II

For cell permeabilization experiments, HeLa cells were incubated with 5 μM rEETI-II-A488 with or without Xfect for 60 min, or transferrin (labeled with Alexa 555, 0.2 mg/ml) for 10 min, at 37 °C/5% CO_2_, washed three times with cold PBS. Cells were then fixed with 4% PFA for 20 min at room temperature, washed three times with PBS, and incubated with either PBS, or detergent containing PBS for 5 min at room temperature. Cells were washed again. To stain nuclei, cells were incubated with Hoechst 333421 (5 µg/ml in PBS) for 10 min and washed three times with PBS then stored in 100 µl PBS in the dark until image acquisition. Fluorescence images were captured on a high throughput ImageXpress Micro XL imaging system (Molecular Devices) and images were analyzed by MetaXpress 4.0. Fluorescence intensity values above a threshold defined using the DMSO-treated samples were measured and used to quantify integrated fluorescence intensity per cell or percentage of dye-positive cells. For endocytic inhibitor treatment, HeLa cells were pre-incubated with nocodazole (10 µM; Sigma, cat # M1404), ikarugamycin (4 μM; Sigma, cat # SML0188), EIPA (50 μM; Sigma, cat # A3085) containing medium for 30 min at 37 °C/5% CO_2_, then incubated with 5 µM rEETI-II-A488 or sEETI-II-A594 (for EIPA only), with or without Xfect, for 60 min in the presence or absence of inhibitors. Cells were washed three times with cold PBS, fixed with 4% PFA for 20 min at room temperature, and washed three times with PBS. Cells were incubated with Hoechst 333421 (5 µg/ml in PBS) for 10 min and washed three times with PBS then imaged, analyzed as described above.

### Electron microscopy

HeLa cells were incubated with rEETI-II-A488, rEETI-II-A488/Xfect or Xfect in T25 flasks as indicated. Cells were fixed with 4% PFA, or 2% PFA + 0.2% glutaraldehyde (GA) in 0.1 M Sorensen phosphate buffer, pH 7.4 for 3 h at room temperature. For PFA-GA fixed samples, cells were washed with PBS for 5 min once. All samples were then stored in 1% PFA in 0.1 M Sorensen phosphate buffer, pH 7.4 until EM analysis. The cells were then embedded in 12% gelatin, cryoprotected with 2.3 M sucrose, mounted on aluminum pins and frozen in liquid nitrogen. Ultrathin cryosections were cut at −120 °C and sequentially incubated on PBS at 37 °C to dissolve gelatin, then at room temperature on antibody solutions, and on Protein A-gold. Final staining of the sections was performed with uranyl acetate (UAc) followed by a UAc-methylcellulose mixture. The antibodies applied were rabbit anti-Alexa488 (Molecular Probes, # A-11094) at 1:50; mouse anti-human LAMP-1 (CD107a) (clone H4A3, BD Pharmingen # 555801) at 1:150; goat anti-human cathepsinD (R&D Systems, # AF1014), at 1:100; rabbit anti-Hrs (M-79; Santa Cruz # sc-30221) at 1:50, and mouse anti-human transferrin receptor (H68.4; Sanbio # 13–6890) at 1:80. Blocking of background labeling was done either with bovine serum albumin (BSA), or a mixture of fish skin gelatin (FSG) and acetylated BSA (BSA-c, from Aurion).

### Biochemical reconstitution of liposomes

Lipids in chloroform were purchased from Avanti Polar Lipids (Alabaster, AL), except cholesterol (powder, Sigma). A dried film was prepared by evaporation of a mixture of the indicated lipids in chloroform. Lipids were hydrated in PBS by five freeze–thaw cycles. The suspension was extruded sequentially 11 times through 0.4 and 0.1 μm (pore size) polycarbonate filters using a hand extruder (Avanti) at a final lipid concentration of 1 mM and used at 100 μM. Liposomes were stored at room temperature, protected from light, and used within 4 days. The composition of the liposomes was 23 mol % 1-palmitoyl-2-oleoyl-*sn*-glycero-3-phosphatidylcholine (POPC), 18 mol % 1-palmitoyl-2-oleoyl-*sn*-glycero-3-phosphatidylethanolamine (POPE), 3 mol % 1,2-dioleoyl-*sn*-glycero-3-phosphoethanolamine-N-lissamine rhodamine B sulfonyl (Rhodamine-PE), 14 mol % liver phosphatidylinositol (PI), 18.5 mol% N-(dodecanoyl)-sphing-4-enine-1-phosphocholine (Sphingomyelin) and 23 mol % cholesterol. For electron microscopy studies, the liposomes were generated without Rhodamine-PE and the amount of POPE was increased accordingly to compensate the liposome composition.

### Liposome quenching assay

Experiments were performed on 96-well plates (100 μl per well) in PBS at room temperature with gentle agitation and protected from light. Liposomes were pre-incubated with rEETI-II, Xfect or both for either 3 or 24 hours. Following pre-incubation, 25 mM TNBS (2,4,6-Trinitrobenzensulfonic acid, Sigma Aldrich) was added to the well and fluorescence was measured using an EnSight plate reader (Perkin Elmer) at 560 nm/580 nm (excitation/emission). Fluorescence signals for samples treated with rEETI-II, Xfect or both were normalized to liposomes alone (control). Data represent averaged (±the standard error of the mean (SEM) of two independent experiments.

### Cryo electron microscopy sample preparation, imaging and processing

3.5 microliters of solution (liposome only, liposome + Xfect, or liposome + rEETI-II) was applied to a freshly glow discharged C-flat holey carbon film with regularly spaced 2 micron holes. The sample was allowed to sit on the grid for 60 seconds then wicked away using Whatman 1 filter paper before additional 3.5 microliters were added to the grid. After 60 seconds incubation, the sample was blotted away for 3.5 seconds and the grids rapidly plunged into liquid ethane using the Vitrobot Mk IV (FEI co.). Grids were then loaded under liquid nitrogen conditions into a Talos F200C (FEI co.) using a Gatan 626 cryo holder (Gatan, Inc). Images were acquired on a CMOS detector (Ceta, FEI co.) at 200 kV using low-dose conditions (~20e/A^2^/image) at a nominal defocus of ~4 microns.

## Supplementary information

Supplementary Info

## Data Availability

No datasets were generated or analyzed during the current study.
