# Supplementary material for: Visualizing the cellular route of entry of a cystine-knot peptide with Xfect transfection reagent by electron microscopy
Source: Sci Rep. 2019 May 6;9:6907. doi: 10.1038/s41598-019-43285-5 (PMC6502800; doi:10.1038/s41598-019-43285-5)
Supplement: Supplementary file 1 — Supplementary Info [file 41598_2019_43285_MOESM1_ESM.pdf]

Supplementary Information

Visualizing the cellular route of entry of a cystine-knot peptide with Xfect transfection reagent by electron microscopy

Xinxin Gao[1], Ann De Mazière[2], David B. Iaea[3], Christopher P. Arthur[4], Judith Klumperman[2], Claudio Ciferri[4] and Rami N. Hannoush[1]*

Departments of Early Discovery Biochemistry[1], Biochemical and Cellular Pharmacology[3] and Structural Biology[4], Genentech, South San Francisco, California
[2]*Section Cell Biology, Center for Molecular Medicine, University Medical Center Utrecht, Utrecht University, Utrecht, The Netherlands*

** To whom correspondence should be addressed: hannoush.rami@gene.com*

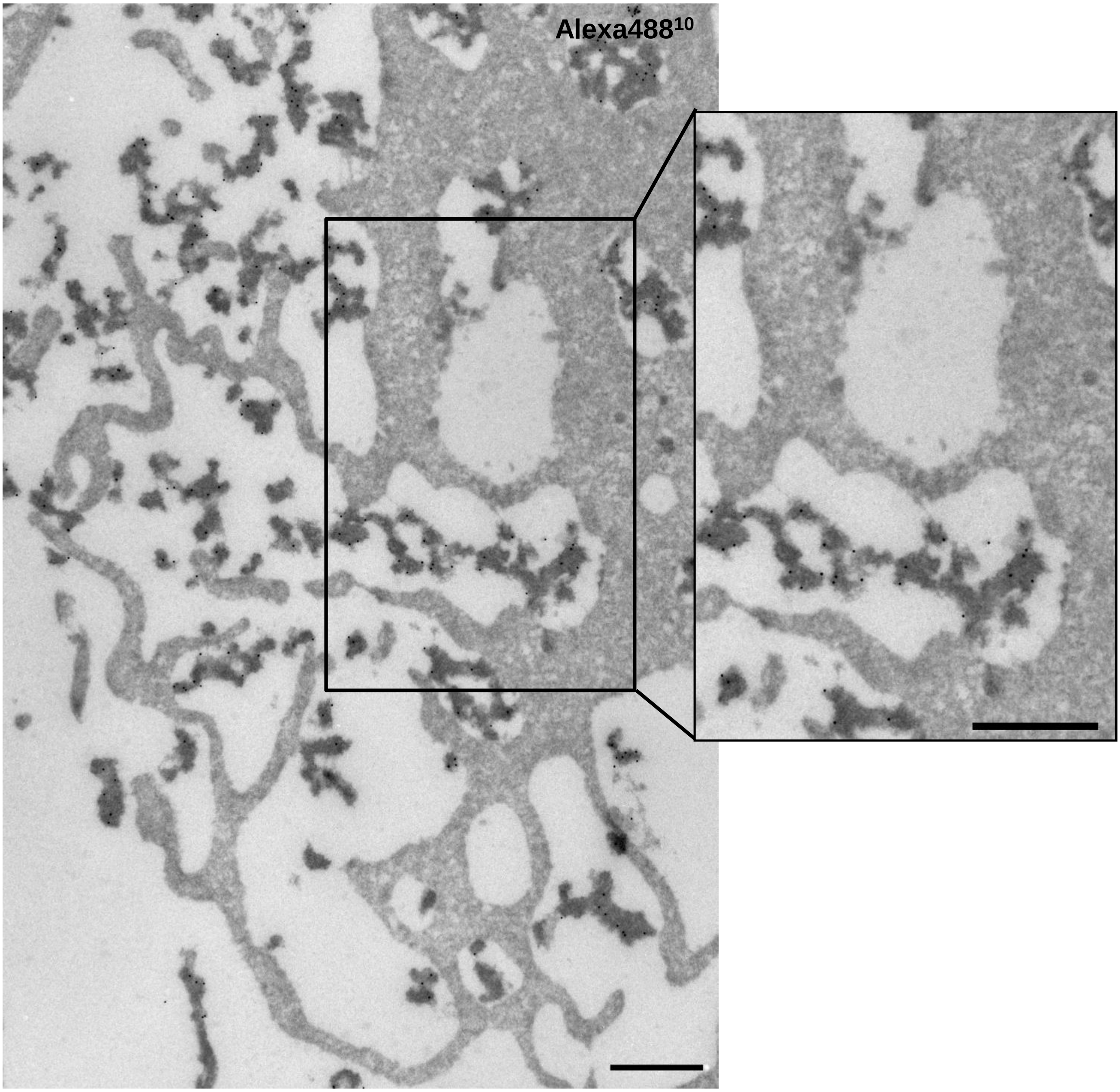

Supplementary Figure 1. Xfect/EETI-II is internalized through macropinocytosis. The image shows membrane ruffles engulfing coagulated rEETI-II-Alexa488. HeLa cells were treated with Xfect/rEETI-II-A488 for 30 min and then fixed with PFA-GA. Scale bar, 500 nm.

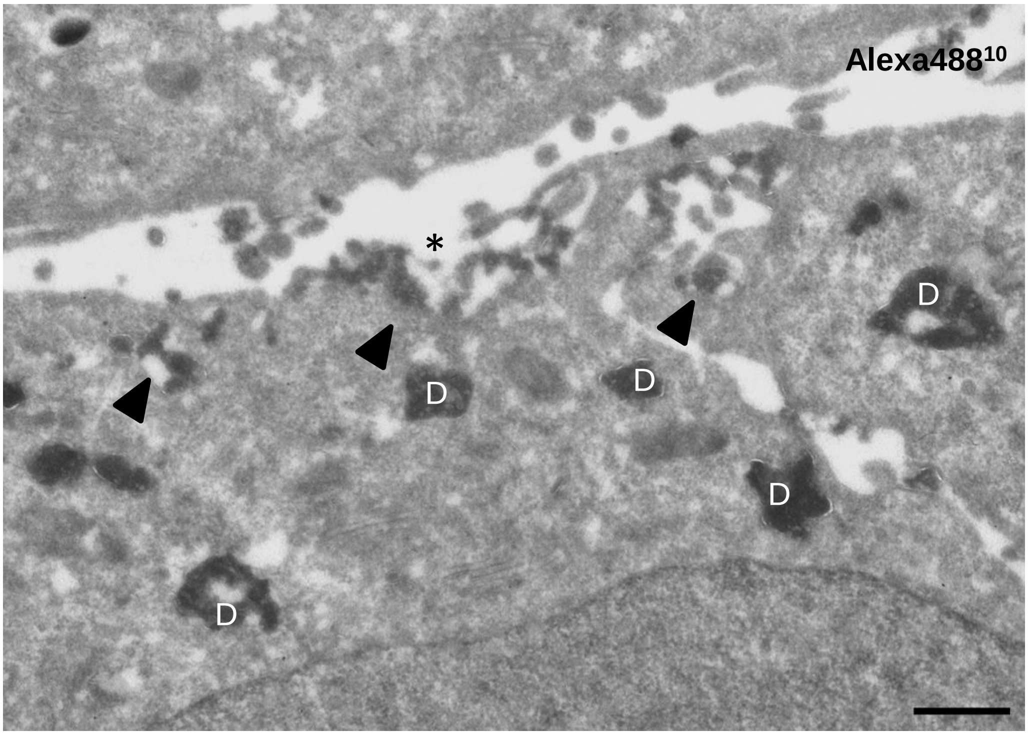

Supplementary Figure 2. Xfect is internalized through macropinocytosis, and forms cellular vesicles similar to those in Xfect/EETI-II. Arrow heads: dense flocculent material (asterisks) apparently in the process of macropinocytosis. D: dense compartments, similar in shape as those found in the samples treated with Xfect/EETI-II. HeLa cells were treated with Xfect for 3 h and then fixed with PFA. Scale bar, 500 nm.

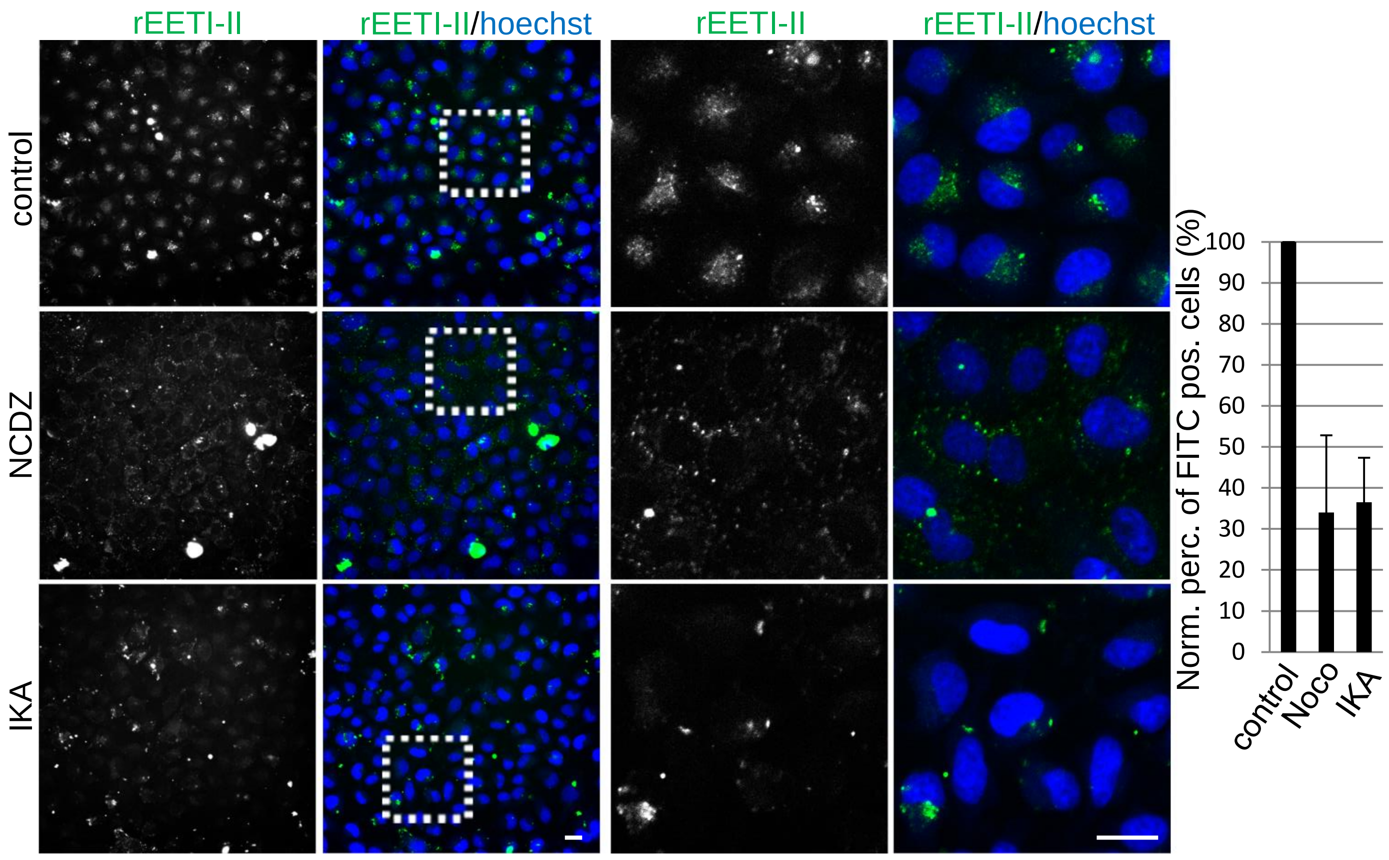

Supplementary Figure 3. Cellular uptake of rEETI-II is reduced upon treatment of cells with nocodazole, an inhibitor of microtubule polymerization, or ikarugamycin, an inhibitor of clathrin-mediated endocytosis. HeLa cells were treated with DMSO, nocodazole (10 µM) or ikarugamycin (4 µM) for 30 min, and then with rEETI-II-A488 (5 µM) in the presence or absence of nocodazole (10 µ M) or ikarugamycin (4 µM) for 60 min at 37 °C. Cells were washed with PBS and then fixed with 4% PFA, imaged and processed as described in methods. Values were normalized to the DMSO-treated samples and averaged from six independent experiments. Mean ± SD. n = 5500 cells. Representative images from six independent experiments are shown. Scale bar, 20 µm.

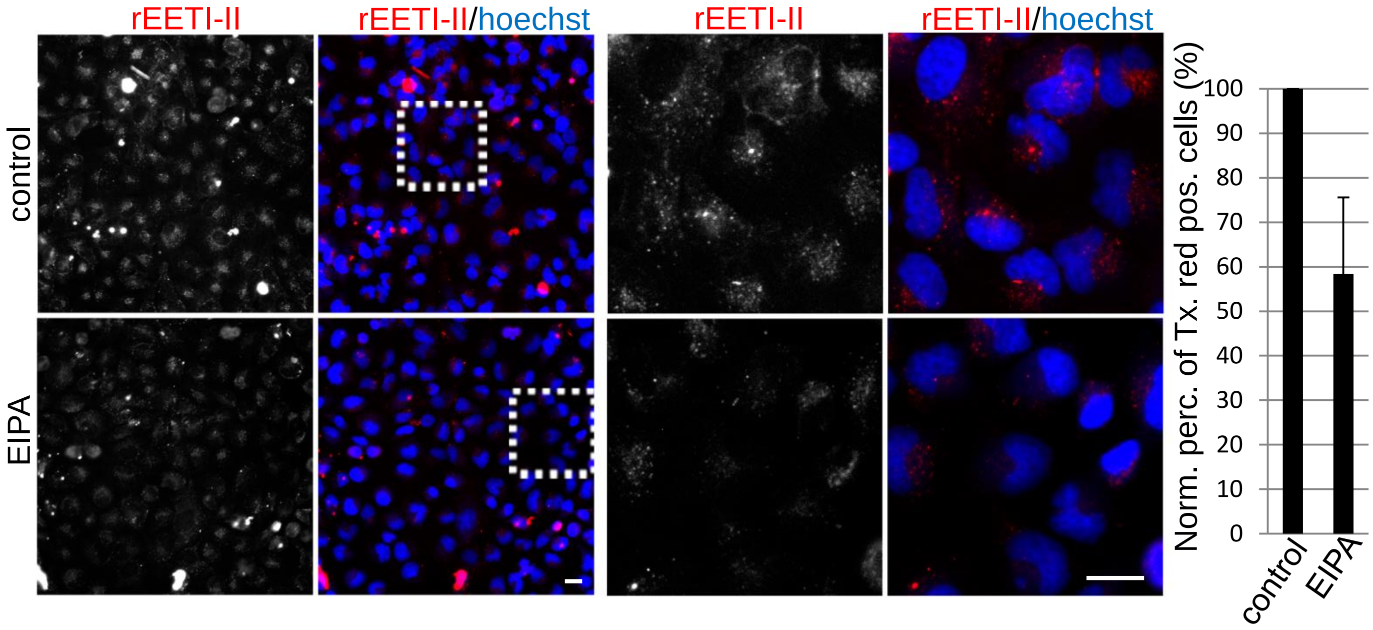

Supplementary Figure 4. Cellular uptake of sEETI-II is inhibited by EIPA, a macropinocytosis inhibitor. HeLa cells were treated with EIPA (50 µM) or DMSO for 30 min, and then with sEETI-II-A594 (5 µM) in the absence or presence of EIPA (50 µM) for 60 min at 37 °C. Cells were washed with PBS, then fixed with 4% PFA, imaged and processed as described in methods. Values were normalized to the DMSO-treated samples and averaged from six independent experiments. Mean ± SD. n = 5500 cells. Representative images from six independent experiments are shown. Scale bar, 20 µm.

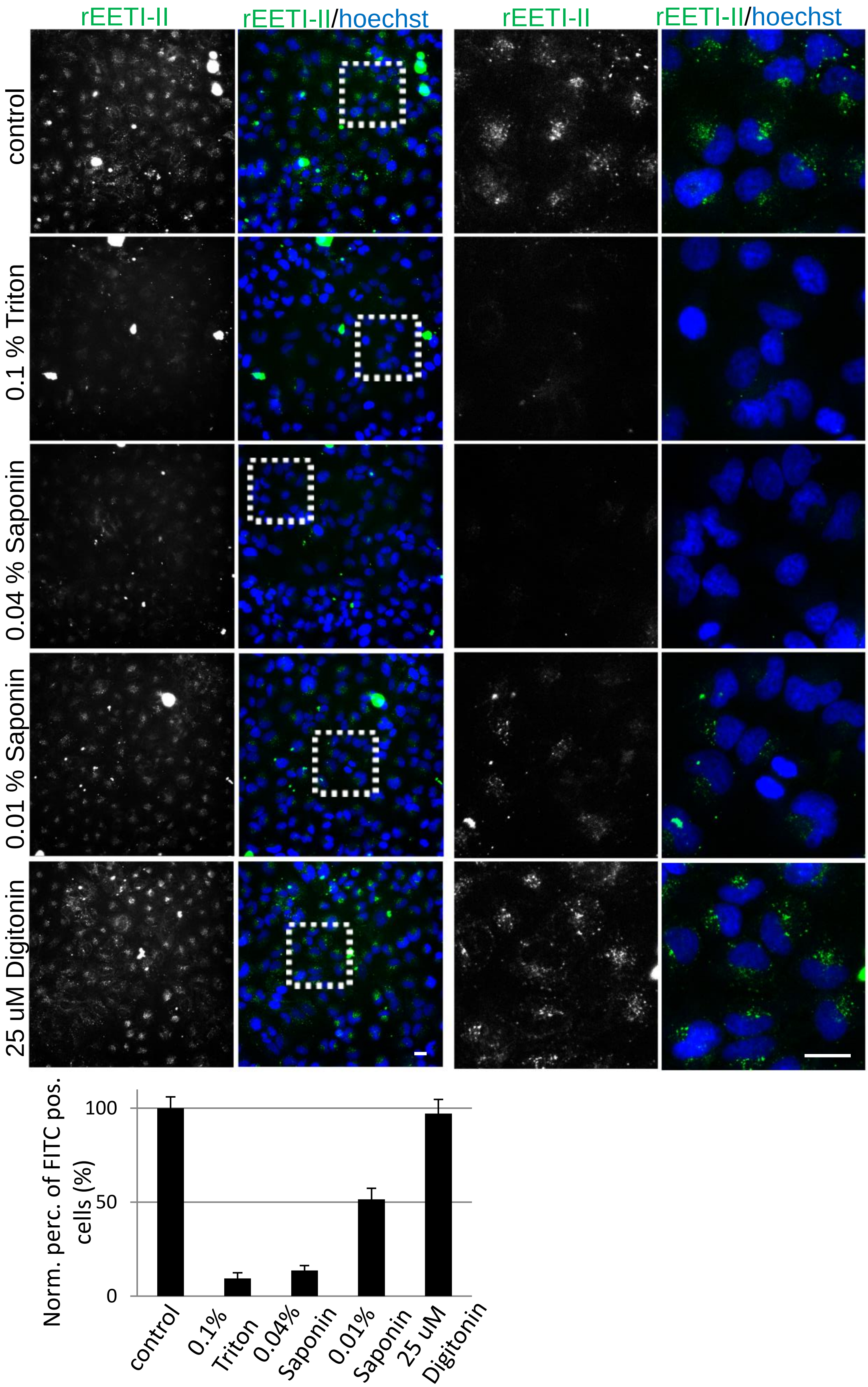
rEETI-II
rEETI-II/hoechst
rEETI-II
rEETI-II/hoechst
control
0.1 % Triton
0.04 % Saponin
0.01 % Saponin
25 uM Digitonin
Norm. perc. of FITC pos. cells (%)
100
50
0
control
0.1% Triton
0.04% Saponin
0.01% Saponin
25 uM Digitonin

Supplementary Figure 5. Internalized rEETI-II localization is sensitive to detergent permeabilization. HeLa cells were treated with 5 µM rEETI-II-A488 for 60 min and then fixed with 4% PFA as described in methods. Cells were then incubated with PBS, 0.1 % Triton X-100, 0.04 % Saponin, 0.01 % Saponin, or 25 µM Digitonin in PBS for 5 min at room temperature, washed and imaged as described in methods. Values were normalized to the PBS-treated samples. Mean ± SD. n = 1000 cells. Representative images from at least three independent experiments are shown. Scale bar, 20 µm.

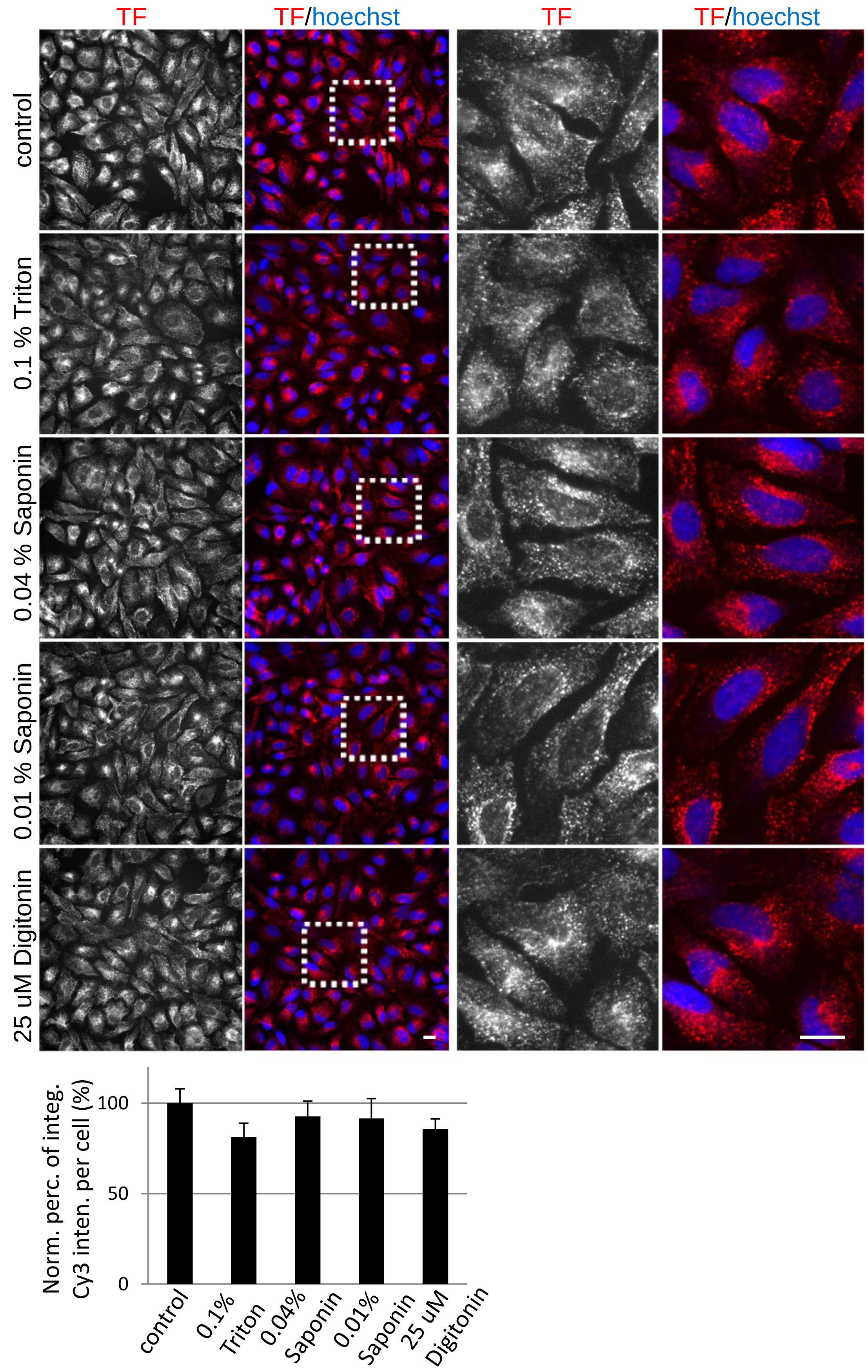
TF
TF/hoechst
TF
TF/hoechst
control
0.1 % Triton
0.04 % Saponin
0.01 % Saponin
25 uM Digitonin
Norm. perc. of integ. Cy3 inten. per cell (%)
100
50
0
control
0.1% Triton
0.04% Saponin
0.01% Saponin
25 uM Digitonin

Supplementary Figure 6. Internalized transferrin maintains its subcellular staining after detergent permeabilization of cells. HeLa cells were treated with 0.2 mg/ml transferrin (Alexa555 labeled) for 10 min and then fixed with 4% PFA as described in methods. Cells were then incubated with PBS, 0.1 % Triton X-100, 0.04 % Saponin, 0.01 % Saponin, or 25 µM Digitonin in PBS for 5 min at room temperature, washed and imaged as described in methods. Values were normalized to the PBS-treated samples. Mean ± SD. n = 1000 cells. Representative images from at least three independent experiments are shown. Scale bar, 20 µm.

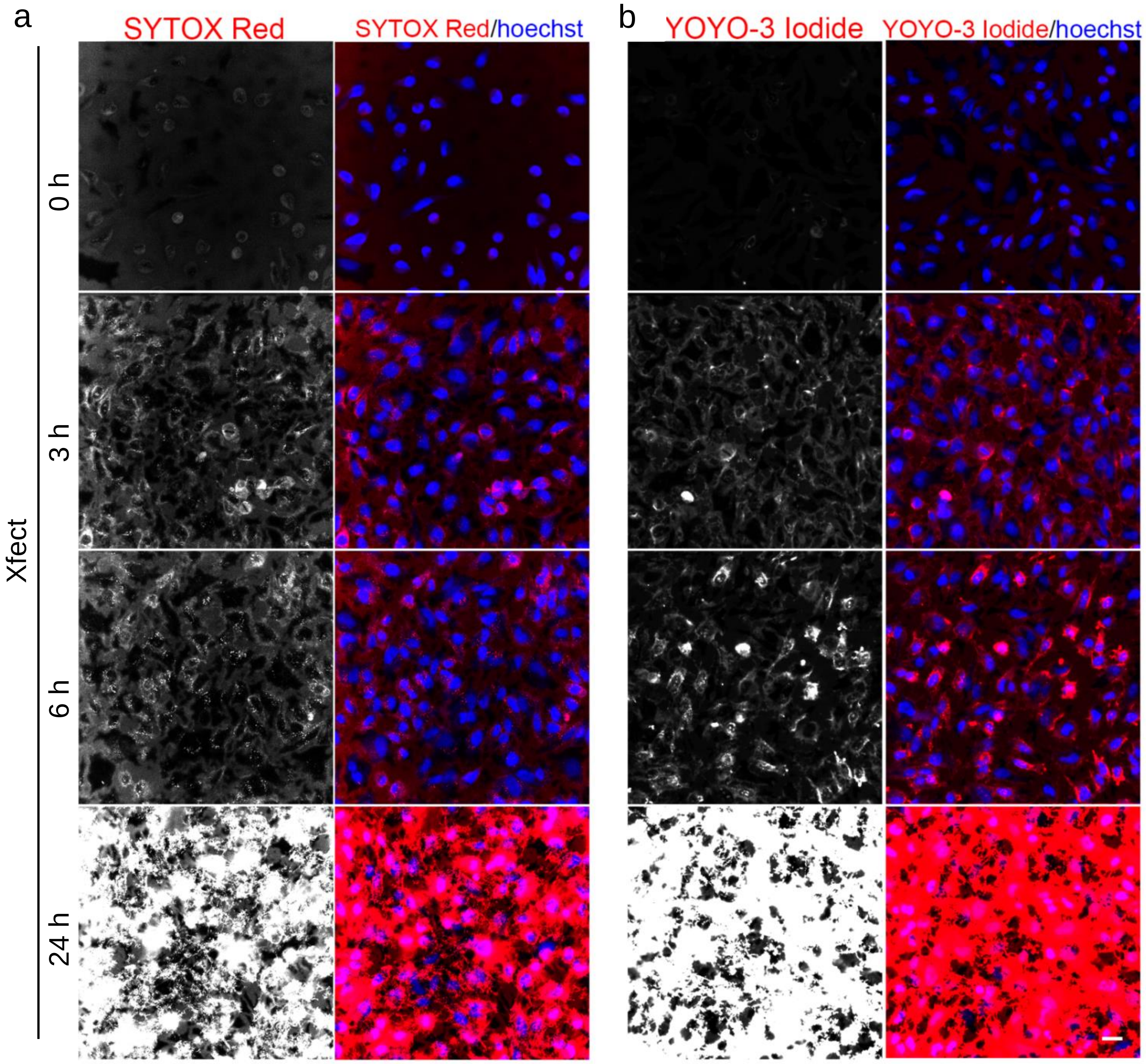

Supplementary Figure 7. Xfect enables the delivery of cell impermeable dyes into HeLa cells. HeLa cells were incubated with Xfect for the indicated times, then washed with PBS for three times and incubated with 30 nM SYTOX Red (a, Cy5 channel) or 200 nM YOYO-3 Iodide (b, Texas Red channel) for 20 min, and washed with PBS again. Cells were fixed with 4% PFA and processed as described in methods. Representative images from three independent experiments are shown. Scale bar, 20 µm. Images are normalized to saturation at the 24 h time point in order to show the cellular distribution of early time point samples.

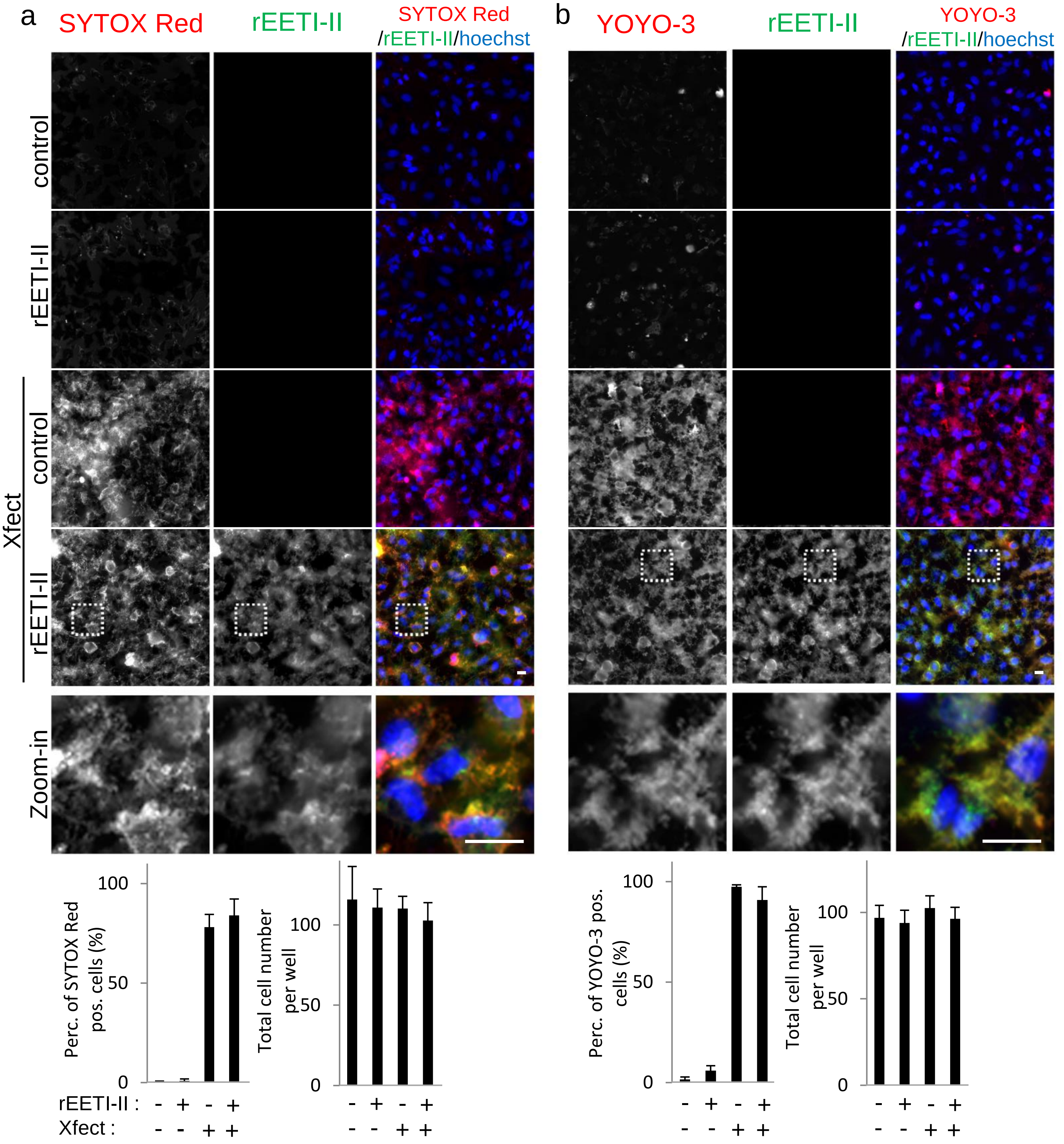

Supplementary Figure 8. Xfect delivers cell impermeable dyes into HeLa cells. HeLa cells were incubated with 5 μM rEETI-II-Alexa488, rEETI-II-Alexa488/Xfect, or Xfect for 24 h. Cells were washed with PBS for three times and incubated with 30 nM SYTOX Red (a, Cy5 channel) or 200 nM YOYO-3 Iodide (b, Texas Red channel) for 20 min then washed with PBS again. Cells were fixed with 4% PFA and processed as described in methods. Mean ± SD. n = 1000 cells. Representative images from three independent experiments are shown. Scale bar, 20 μm.
